# Distribution of Major Basic Protein on Human Airway following In Vitro Eosinophil Incubation

**DOI:** 10.1155/2010/824362

**Published:** 2010-03-22

**Authors:** Ailing Xue, John Wang, Gary C. Sieck, Mark E. Wylam

**Affiliations:** ^1^Division of Pulmonary and Critical Care Medicine, Department of Medicine and Pediatrics, Mayo Clinic College of Medicine, Rochester, MN 55905, USA; ^2^Department of Physiology & Biomedical Engineering, Mayo Clinic College of Medicine, Rochester, MN 55905, USA

## Abstract

Major basic protein (MBP) released from activated eosinophils may influence airway hyperresponsiveness (AHR) by either direct effects on airway myocytes or by an indirect effect. In this study, human bronchi, freshly isolated human eosinophils, or MBP purified from human eosinophil granules were incubated for studying eosinophil infiltration and MBP localization. Eosinophils immediately adhered to intact human airway as well as to cultured human airway myocytes and epithelium. Following incubation 18–24 h, eosinophils migrated into the airway media, including the smooth muscle layer, but had no specific recruitment to airway neurons. Eosinophils released significant amounts of MBP within the airway media, including areas comprising the smooth muscle layer. Most deposits of MBP were focally discrete and restricted by immunologic detection to a maximum volume of ∼300 *μ*m^3^ about the eosinophil. Native MBP applied exogenously was immediately deposited on the surface of the airway, but required at least 1 h to become detected within the media of the airway wall. Tissue MBP infiltration and deposition increased in a time- and concentration-dependent manner. Taken together, these findings suggest that eosinophil-derived cationic proteins may alter airway hyperresponsiveness (AHR) in vivo by an effect that is not limited to the bronchial epithelium.

## 1. Introduction

Asthma is a chronic inflammatory disease of the airways, which reduces airway luminal diameter by several mechanisms, including increased airway mucus, airway edema, and airway smooth muscle activation. A histologic hallmark of clinical and experimental asthma is the prevalence of eosinophils within, and infiltrating, the airway wall [1, 2]. In asthma deaths, the eosinophilic inflammation is more intense than in mild-to-moderate asthma [3]. However, in peripheral airways the eosinophilic infiltration is significant even during atopic asthma in remission [4, 5]. The presence of airway eosinophils in asthma is in stark contrast to their near total absence in healthy persons. Eosinophil granules contain proteins, including major basic protein (MBP), which are highly basic and cationically charged and may influence airway cell function and cause AHR [6]. Recently, the role of the eosinophil in asthma has been questioned as anti-IL-5, which effectively depleted eosinophils from the blood and induced sputum in mild atopic subjects with asthma, but had no effect on AHR [7]. However, in similar studies, anti-IL-5 also had no effect on bronchial mucosal staining of intracellular and extracellular MBP [8]. In these studies the MBP deposition was only evaluated at the level of the airway mucosa. Thus, the lack of efficacy of anti-IL-5 on AHR via granule protein inhibition was incompletely understood.

**Figure 1:**
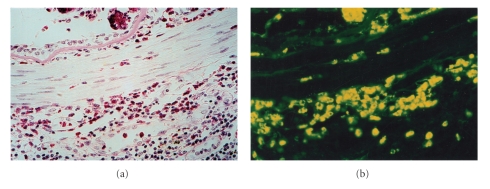
(a) Example of intense inflammatory infiltration of eosinophils into the lamina propria in airway of asthmatic patient (H&E stain; 600x). Note discrete localization of eosinophils within the ASM layer. (b) Example of immunofluorescence staining of MBP in the ASM layer of an asthmatic patient (anti-MBP; 600x). In addition to the lamina propria, MBP was clearly localized in the smooth muscle layer.

Prior investigations, both in vivo [9, 10] and in vitro [11–14], using direct, but brief, applications of purified MBP have suggested that the effect of MBP on smooth muscle cell contraction is *indirect* and likely mediated via barrier interruption of the airway epithelium or by altering epithelial mediator release [15]. However, these prior investigations rest on the hypothesis that cationic peptides delivered to the airway luminal surface *mimic* in vivo eosinophil granule protein release and that granule protein release never occurs within the airway wall. An analysis of the extracellular deposition of granule proteins within the airway wall has been conducted in guinea pig models of asthma. In those studies, “M_2_ receptor dysfunction” is “restored” by an antibody to MBP [16], or by the MBP antagonist heparin sulfate [17]. These results suggest that extracellular MBP deposition does occur within the airway wall. However, these studies leave open the possibility of a direct effect of MBP on airway myocytes. In an index case of a patient who died of status asthmaticus, we identified eosinophils, as well as extracellular MBP within the smooth muscle layer (Figures 1(a) and 1(b)). Moreover, we have previously demonstrated in cultured airway myocyte preparations that MBP *directly* alters cellular calcium homeostasis, suggesting the possibility that MBP may *directly* augment smooth muscle contraction [18]. It is possible that airway preparations are relatively impermanent to the direct application of MBP. Several investigators have qualitatively noted MBP within the airway wall of patients with asthma [4, 5, 19, 20]. But, in none of these studies was an analysis of the MBP performed such that the granule protein localization could be differentiated between intracellular and extracellular deposition, nor was a precise localization of the eosinophils in relative distance and distribution to myocytes and airway neurons established. Thus, the physiologic effects of MBP on human airway smooth muscle remain uncertain.

As MBP is transported directly to individual airway cells following eosinophil migration, activation, and degranulation, we sought to understand the potential role of MBP on airway cells by determining the spatial localization of both MBP and eosinophils following direct application of either purified MBP as well as following its in vitro release from human eosinophils.

## 2. Methods

### 2.1. Human Bronchi Isolation

Human bronchi were obtained from surgical specimens in accordance with procedures approved by the Mayo Clinic Foundation Institutional Review Board. Tissues obtained were incidental to the patient's surgery and were discarded by the surgical pathologist. The total subjects were 22, airways from 15 subjects were incubated with eosinophils, and airways from 9 subjects were incubated with MBP. All tissues were immersed in ice-cold HBSS (Hanks balanced salt solution with 2.25 mM CaCl_2_, 0.8 mM MgSO_4_ and 12, mM glucose; pH 7.4). 3rd to 6th generation bronchi were freed from adherent tissue using a dissecting microscope. Bronchial segments (4-5 mm length, 1-2 mm diameter) were prepared and kept in ice-cold oxygenated HBSS until use in experimental protocols. For incubation studies bronchial segments were washed thoroughly and transferred to eight-well Lab-Tek glass cover slides (Nunc, Naperville, IL) in DMEM supplemented with 10% FBS and antibiotics/antimycotic (penicillin, streptomycin, and amphotericin B) and maintained in a 5% CO_2_ humidified incubator at 37°C.

### 2.2. Human Bronchial Smooth Muscle and Epithelial Cell Isolation and Culture

Human bronchi were obtained and epithelium was removed as described above for use in human bronchi isolation. Primary smooth muscle cultures were prepared as previously reported [21]. Briefly, third- to sixth-generation bronchi were immersed in icecold HBSS aerated with 100% O_2_. Under a dissecting microscope, epithelium was removed, and the remaining tissue was finely minced in icecold Ca^2+^-free HBSS. The HBSS was removed, and the tissue was incubated for 1 h at 37°C in Earl's balanced salt solution (EBSS) containing 30 mg/mL BSA, 20 U/mL papain, and 0.005% DNase (Worthington Biochemical, Lakewood, NJ). After 1 h, 1 mg/mL of type IV collagenase and 0.4 U/mL elastase were added for an additional 45 min at 37°C. The tissue was gently triturated, centrifuged, and redispersed several times in EBSS with final resuspension in DMEM/F-12 medium containing antibiotics/antimycotic and 10% fetal bovine serum. The final resuspension was seeded into culture flasks. 

All cultures were maintained in a humidified atmosphere of 5% CO_2_ and 95% air at 37°C. Upon reaching 70%–90% confluence, cells were passaged and plated for use in experiments. Cells were used between passage 1 and 2 for all experiments. Presence of smooth muscle cells was confirmed by immunohistochemistry. For epithelial cell isolation, bronchial segments were dissected clean of adherent connective tissue and placed (with both ends ligated) into sterile HBSS. The lumen was filled with 0.1% Protease (Sigma) for 1 to 2 hr at 37°C. Subsequently, cells were mechanically loosened from the airway lumen with a rounded spatula suspended in HBSS for centrifugation at 600 rpm. The resulting cell pellet was then washed (twice) and resuspended in HBSS with 10% FCS. Cells were seeded on to Type IV collagen coated glass cover slides in F12 with epithelial growth factor, Triiodothyroxine, penicillin and 10% FCS and grown to confluence in 2–4 days.

### 2.3. Isolation of Human Eosinophils

Human eosinophils were isolated as previously described [22]. Briefly, heparinized blood was collected from atopic and nonatopic volunteers, and equal volume of 1 × PIPES was added, and the diluted blood was layered onto Percoll (density 1.085 g/mL). After centrifugationat 1000 g for 30 minutes at 4°C, the plasma and Percoll layers were removed by aspiration. Tubes were wiped to remove contaminating leukocytes, and red cells were lysed by osmotic shock. The remaining pellet, containing neutrophils and eosinophils, was incubated with an equal volume of anti-CD16 magnetic beads (Miltenyi Biotec, Auburn, CA) on ice for 30 minutes. After incubation the cell mixture was diluted with 1 × PIPES + 1% calf serum (HyClone Laboratories, Logan, UT) and eluted through a steel wool column suspended in a strong magnet. Column eluate (14 mL) was collected and the number of eosinophils was determined by staining with Randolph's stain.

### 2.4. Isolation Human MBP

Eosinophils as isolated above were lysed with sucrose and heparin, and the granules were collected by centrifugation. Granules were lysed by exposure to 0.01 M HCL, pH 2, and by sonication. Insoluble materials were removed by centrifugation and the lysate was applied to a Sephadex G-50 column, equilibrated with 0.025 M sodium acetate buffer, with 0.15 M NaCl, pH 4.2. The third peak fractions were pooled as MBP [23].

### 2.5. Incubation of Bronchial Segments or Airway Cells with Eosinophils or MBP

Freshly isolated eosinophils were added to wells (final volume of media = 400 *μ*L per well) which contained bronchial segments or cultured airway myocytes and epithelium. The final concentration of isolated eosinophils in all groups was 2.5 × 10^6^ cells/mL. After 10 min and during direct video microscopy nonadherent eosinophils were removed by washing 3 times with fresh media. Using a calibrated optical grid, the number of adherent cells remaining was counted and recorded. Airway smooth muscle cultures were fixed in 10% formalin for 2 min at room temperature and eosinophils were identified using Wright's stain. 

For prolonged incubation, to determine the localization of eosinophils and/or immunoreactive MBP, bronchial segments were exposed to either eosinophils or MBP, and incubated for 24 h in medium (400 *μ*L) ± eosinophils (2.5 × 10^6^ cells/mL) + IL-5 (100 pg/mL) for 18 h. IL-5 was added to the medium as it has previously been determined necessary to prevent eosinophil apoptosis in vitro [24]. In bronchi incubated with eosinophils 4 h prior to fixation, 25 ng/mL of IL-5 was added for additional 4 h to promote eosinophil degranulation [25]. In bronchial segments incubated with purified MBP (10^−9^–10^−5^ M) the incubation time varied between 1 min, 1 h, or 24 h. Following incubation tissues were fixed and immunostained as described below.

### 2.6. Histology and Fluorescent Immunohistochemistry for Analysis of Eosinophils Location and Immunoreactive MBP

First to identify and quantify the possibility of migration of eosinophils into bronchial segments in vitro, histologic stains were used. Specifically, to identify eosinophils following overnight incubation of bronchial segments with eosinophils, segments were imbedded in Tissue-Freezing Medium (Triangle Biomedical Sciences, Durham, NC) and snapfrozen in isopentane precooled in liquid nitrogen. 10 *μ*m serial sections were then cut and stained with hematoxylin and eosin (H&E) for conventional histologic examination; additional sequential sections were stained for eosinophils with Wright's stain. Only “unambiguous” cells with eosin containing granules were identified as eosinophils and quantified per cross-sectional area. Eosinophils were identified as within the smooth muscle layer if all, or part of the cell, contacted an unambiguous smooth muscle cell. We then quantified the number of eosinophils within the smooth muscle layer. As histologic stains are less sensitive than immunostaining for eosinophils, we likely undercounted the total number of eosinophils which migrated into tissues compared to control tissues. 

Other bronchial sections were immunostained for MBP and smooth muscle actin. Filley et al. have shown that the antibody is specific for MBP because it stained only eosinophils in the peripheral blood and because its activity is removed by absorption with MBP [26]. Briefly, sections were fixed in ice-cold acetone: methanol (60  : 40) for 10 min, washed with 0.3% triton x –100 in tris buffered saline (TBS) with tween 20 (TBST; pH 7.6), and air dried. Sections were blocked in 10% donkey serum in TBST for 1 h, 1% chromotrope 2R to block nonspecific binding of fluorescein dye to the eosinophils [27]. Sections were then incubated with the primary antibodies (MBP 1  : 60, actin 1  : 500, protein gene product (PGP) 1  : 200 alone, or in combinations) diluted in 1% donkey serum in TBST for 2 h at room temperature. Control sections were treated identically in the absence of primary antibodies. After incubation with the primary antibodies, the sections were rinsed with TBST and treated with Cy3 (1  : 200) and Cy5 (1  : 500) conjugated species, specific secondary antibodies for 45 minutes at room temperature. MBP and actin immunoreactivity were imaged with an Olympus FluoView confocal microscope mounted on a BX50WI microscope (Olympus America, Melville, NY). An Olympus Dapo × 40/1.3 NA oil-immersion objective was used for imaging. Serial confocal optical sections were obtained by moving the stage in only one direct. Each optical section was digitized and stored in arrays of 800 × 600 pixels. Pixel dimensions were calibrated using a stage micrometer and were found to be 0.5 × 0.5 *μ*m for the *x* *y*-plane. The calculated thickness of optical sections was matched to this dimension such that each voxel was 0.25 *μ*m^3^. These values were used to calculate the volume of MBP, which surrounded degranulated eosinophil “ghosts.” 

On some images computer analysis of sections stained for MBP by immunofluorescence was performed to objectively evaluate the overall areas covered by MBP and smooth muscle. Digital images obtained above were selected for maximal fluorescence staining of either MBP or actin. Imaging and acquisition parameters were initially determined to verify lack of photobleaching or visible photodamage. Digital images of the next section in a series which were stained with normal rabbit IgG served as a negative control and were recorded. A threshold setting was acquired for each negative control image so that pixel values were near zero and images and subsequently positively stained images were acquired at these settings. Both camera background (dark current) and image background (taken from fluorescent image signal collected from random 10–12 ROIs of the negative control specimens) were determined daily and subtracted from the collected quantified image fluorescence. In addition, xenon lamp intensity, image acquisition time, and image pixel binning were fixed and controlled such that all image signals remained within the 12 bit dynamic intensity range of our digital camera, and thus could be reliably compared across experiments. We identified MBP as being present at a tissue site (voxel) if the florescent signal was >5% above background in our 4095 dynamic range 12 bit imaging system. This value was chosen because maximal florescent intensity in tissues treated with exogenous MBP (10^−5^ M) typically gave intensity values of ~2500 following image correction. Given these constraints, we could only underestimate, but never overestimate, the presence of MBP. Later images were deconstructed into an image specific for smooth muscle actin and one specific for immunoreactive MBP using Metamorph (Molecular Devices, Sunnyvale, CA, 94089). Subsequently these two images were masked and binarized so that the percentage of actin specific smooth muscle and MBP could be determined as well as the relative area that was colabeled.

### 2.7. Quantification of Eosinophils and Extracellular MBP in the Vicinity of PGP 9.5 Positive Airway Neurons

Following overnight incubation of 2 mm human bronchi with eosinophils, histological sections were prepared and double immunostained with nonspecific neuronal cell marker anti-PGP 9.5 and anti-MBP. A 10 *μ*m grid was overlaid onto images collected with 400X magnification. All squares within sections of each airway were counted as containing intact eosinophils, extracellular MBP, PGP 9.5 neurons, or combinations, or none of above. Percentage of squares containing PGP 9.5 and eosinophils or extracellular MBP were determined and compared to the percentage of squares not containing PGP 9.5 neurons.

### 2.8. Determination of Eotaxin Presence in Unstimulated Human Airway by Fluorescent Immunohistochemistry

Because we found exogenously added donor eosinophils migrating within unstimulated human airway following incubation, we determined whether expression of EOTAXIN was present by immunostaining as above. Serial sections were immunostained for MBP and Eotaxin, as above (MBP 1 : 60, Eotaxin 1 : 50), and diluted in 1% donkey serum in TBST for 2 h at room temperature. Control sections were treated identically in the absence of primary antibodies. After incubation with the primary antibodies, the sections were rinsed with TBST and treated with Cy3 (1 : 200) and Cy5 (1 : 200) conjugated species, specific secondary antibodies for 45 minutes at room temperature. MBP and Eotaxin immunoreactivity were imaged with an Olympus FluoView confocal microscope mounted on a BX50WI microscope (Olympus America, Melville, NY).

## 3. Materials

Human eosinophil-derived MBP in Na acetate buffer (pH 4.0, ~1–3 mg/mL) and human eosinophils were obtained from the laboratory of Dr. Hirohito Kita (Mayo Clinic, Rochester, MN). DMEM and antibiotic/antimycotic mixtures were obtained from Gibco-Invitrogen, Carlsbad, CA. Interleukin-5 was a gift from Schering-Plough, Kenilworth, NJ. Mouse monoclonal antibody for MBP, and goat polyclonal antibody for actin, goat polyclonal antibody for Eotaxin were purchased from Santa Cruz Biotechnology (Santa Cruz, CA). Rabbit polyclonal antibody for PGP 9.5 was purchased from Biogenesis. Cy3-conjugated affinity-purified donkey anti-mouse IgG and cy5-conjugated affinity-purified donkey anti-goat and anti-rabbit IgG were purchased from Jackson ImmunoResearch Laboratories, Inc. (West Grove, PA). Human plasma fibronectin was purchased from Gibco-Invitrogen Corporation, Carlsbad, CA. Collagen was purchased from Vitrogen 100, Cohesion, Palo Alto, CA. Bovine serum albumin was purchased from Sigma-Aldrich, St. Louis, MO.

## 4. Data Analysis

In all experiments, differences between two groups were analyzed for statistical significance using either a one-way analysis of variance (ANOVA) or a Student's *t*-test (two-tailed) for unpaired sample. In the case of ANOVA, a multiple-comparison test was used to compare all groups. A value of *P* ≤ .05 was accepted as significant. All results are expressed as mean ± S.E.

## 5. Results

### 5.1. Patient Characteristics

Using established guidelines [28], subjects were classified into normal lung function, or mild, moderate, and severe COPD. Specific characteristics of each group including smoking history are listed in Table 1. Among the 22 subjects, 17 had bronchogenic carcinoma; the other 5 had bronchoalveolar carcinoma (1), carcinoid (1), metastatic sarcoma (1), mycetoma (1), and bronchopleural fistula (1).

### 5.2. Identification by Immunofluorescence of MBP in Smooth Muscle Layer of a Patient with Asthma

In formalin-fixed, paraffin-embedded lung tissue obtained post mortem from a patient who died due to asthma (Figure 1(a)), indirect immunofluorescence (Figure 1(b)) showed areas of extracellular MBP immunofluorescence within the smooth muscle layer. These areas showed few intact eosinophils in a sea of eosinophilic granular debris, which stained intensely with anti-MBP. These results strongly suggest that in addition to necrotic areas in the lamina propria and damaged epithelial surfaces, in this one patient with asthma, MBP was released within the smooth muscle layer of the airway.

### 5.3. Eosinophils Immediately Adhere to Human Airway In Vitro and Cultured Human Airway Myocytes and Epithelium

**Figure 2:**
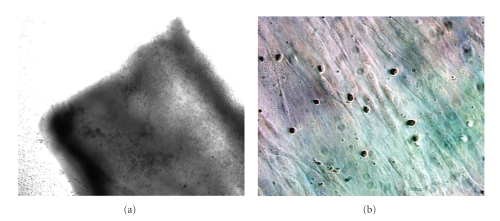
(a) Human eosinophils immediately (<10 min) adhere to freshly isolated human airway in vitro. Human eosinophils adhere to adventitial surface of human airway and epithelial surface (not seen) (bronchial diameter = 1.5 mm, phase contrast 100x). (b) Eosinophils immediately (<10 min) adhere to cultured human airway myocytes. Wright's stain of eosinophils adhering to passage 1 serum-deprived elongated airway myocytes (phase contrast 600x).

Repeated observations by phase contrast microscopy (*n* = 3 bronchial segments from 5 distinct patients) revealed that exogenously applied unstimulated eosinophils adhered immediately (<10 min) to the adventitial and epithelial surface of 2 mm human airways (Figure 2(a)) as well as to the surface of the glass chamber. Within 10 minutes the attachment appeared durable, as direct rinsing of the tissue with fresh media elicited no discernible detachment of eosinophils. Similarly, exogenously applied unstimulated eosinophils adhered immediately (<10 min) to surfaces of cultured (passage 1-2) human airway myocytes (Figure 2(b)) and primary human airway epithelium (not shown). Similar to fresh tissue, the attachment was durable, as direct rinsing of the tissue with media during observed microscopy showed no discernible detachment of eosinophils from the cells. Immediate eosinophil adherence to cultured ASM and epithelium was 10 ± 3, 20 ± 5%, respectively, *n* = 3 determinations from Wright's stained cover slides of ASM cells from 5 distinct patients of the eosinophils added to the culture system. The attachment was Ca^2+^ dependent as media with 0.0 mM Ca^2+^ and 0.3% EDTA completely prevented eosinophil attachment.

### 5.4. Eosinophils Migrate within the Airway Wall, Including the smooth Muscle Layer

**Figure 3:**
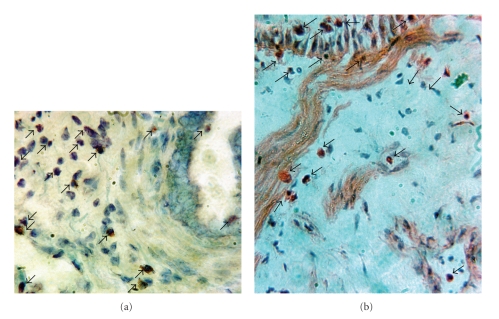
Following overnight incubation adherent eosinophils migrated within the airway wall, including the smooth muscle layer. Eosinophils were found within the epithelium, subepithelium, smooth muscle layer, and loose connective tissue. These panels represent examples of eosinophils identified by Wright's staining (a) and H&E staining (b) within the airway wall; arrows denote eosinophils (600x).

Following eosinophil incubation of small human airways for 18–24 h, eosinophils, identified by Wright's or H&E stain, migrated within the airway wall, including into the smooth muscle layer (Figures 3(a) and 3(b)). The total numbers of eosinophils found within the airway were 557 ± 68 per cross-section of ~2 mm airways (*n* = 3 airway sections from 11 distinct patients). The total numbers of eosinophils found within or adjacent to the airway smooth muscle layer were 124.9 ± 18.1 per cross-section of ~2 mm airway (*n* = 5 airway sections from 5 distinct patients). In tissues incubated without eosinophils (control tissues), total eosinophils within the entire airway were 3 ± 1 and eosinophils within or adjacent to the airway smooth muscle layer were 0 ± 0 per cross-section of ~2 mm airway (*n* = 3 airway sections from 11 distinct patients in both groups).

### 5.5. Distribution of Immunoreactive MBP and Smooth Muscle Actin in Human Airway Bronchus following Overnight Incubation with Human Eosinophils

**Figure 4:**
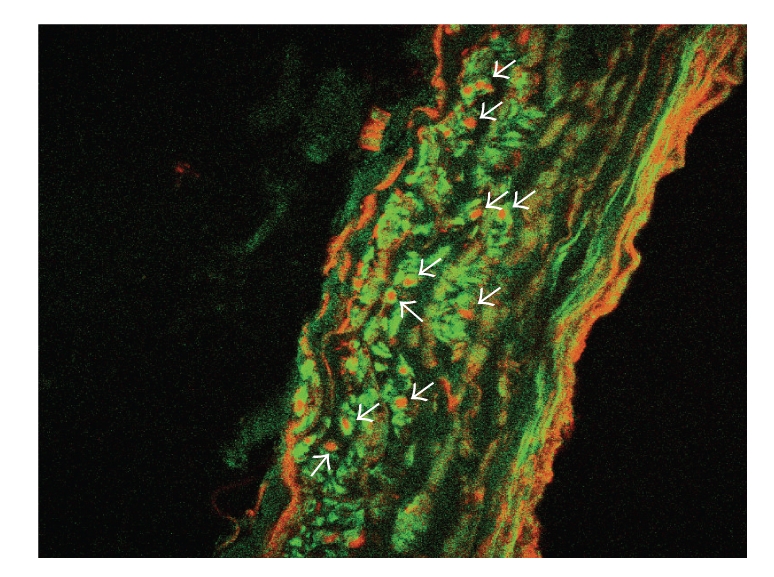
Representative image showing distribution of immunoreactive MBP and smooth muscle actin within 2 mm human airway following overnight incubation with human eosinophils. Immunoreactive actin (green) and MBP (red) are shown. Two patterns of extracellular MBP deposition are noted broad linear deposition along luminal and abluminal surfaces, and (arrows) discrete focal deposition within the airway media, especially in the vicinity of airway myocytes (confocal microscopy 400x).

Following overnight incubation with eosinophils, immunoreactive MBP was demonstrated over 16.8 ± 3.2% (*n* = 5 airway sections from 15 distinct patients) of the airway volume (Figure 4). Using immunoreactive smooth muscle actin to determine the location of airway myocytes, binarized colabeled images demonstrated that 23.8 ± 4.9% (*n* = 5 airway sections from 15 distinct patients) of the total MBP content was resident in a smooth muscle specific locus. Eosinophil degranulation was evident by two patterns of extracellular MBP deposition. A broad linear deposition occurred along both the adventitial and epithelial surface. In addition confocal scanning microscopy determined that the MBP deposition within the media occurred as discrete focal deposition occurring within the airway media, including the airway myocyte. These focal deposits of MBP were demonstrated as both intracellular MBP as well as extracellularly released MBP (vide infra). In all control tissues no MBP content was observed.

### 5.6. Microdomain of MBP Release from Individual Eosinophils

**Figure 5:**
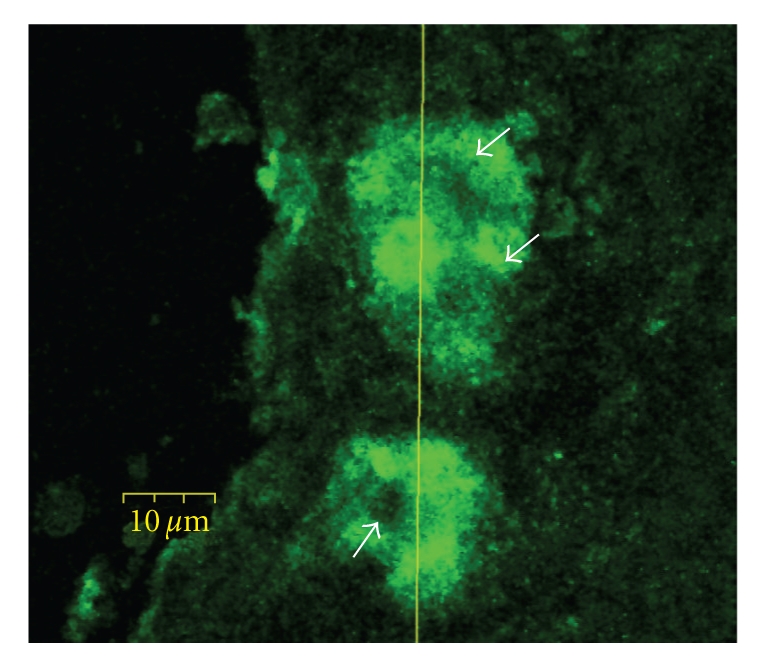
Distribution of immunoreactive MBP domain within human airway following overnight incubation with human eosinophils. MBP (green) is shown as focal deposits. Arrows indicate eosinophil “ghosts” (confocal microscopy 600x).

Confocal laser scanning of areas of MBP staining within the airway media demonstrated both intracellular MBP as well as restricted extracellular microdomains of MBP with “ghosts” representing degranulated eosinophils (Figure 5). The immune detected microdomain of MBP released from individual eosinophils within the human airway was observed to be restricted to an average of 317.5 ± 43.5 *μ*m^3^ from the center of a ghost eosinophil within the airway (*n* = 5 determinations each per 10 patients). Similarly the microdomain of MBP released from individual eosinophils at sites along the perimeter of the airway (either luminally, or abluminally) was restricted to an average of 628.5 ± 213.9 *μ*m^3^(*n* = 5 determinations each per 10 patients).

### 5.7. Distribution of Immunoreactive MBP and Smooth Muscle Actin in Human Airway Bronchus following Exogenously Applied MBP in Human Airway

**Figure 6:**
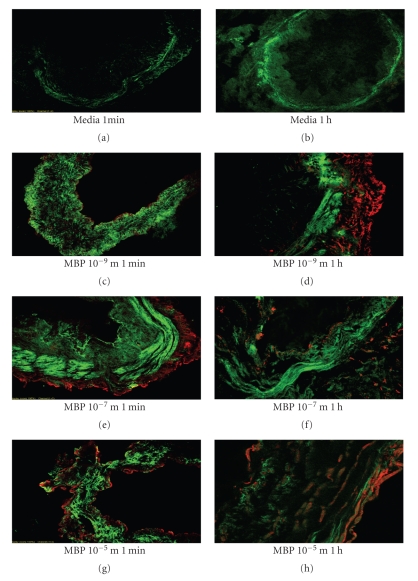
Immunolocalization of exogenously applied MBP in human airway. Results show representative images analyzing the fluorescence of immunolabeled MBP in human airway following incubation with MBP for 1 min and 1 h. Immunoreactive actin (green) and MBP (red) are shown. *Brief Exposure*. 1 min elicited limited MBP deposition to the epithelial and outer surfaces of the airway. However, longer 1 h exposure allowed some MBP to access cells within the media of the airway wall. The longer 1 h exposure was necessary for immunolocalization to be detected within the smooth muscle layer. This time-dependent effect was increased by greater concentrations of MBP (10^−5^ M) compared to lesser concentrations of MBP (10^−7^ M). In all control tissue no MBP content was detected (confocal microscopy 400x).

**Figure 7:**
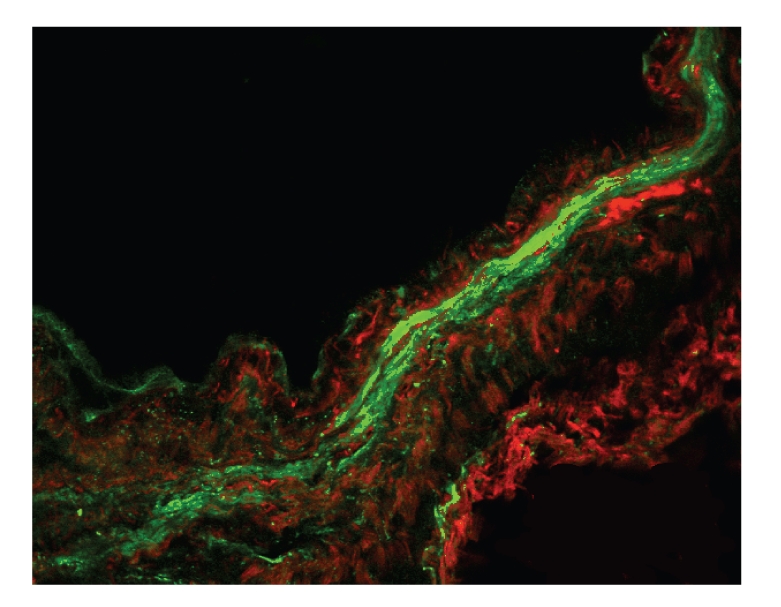
Overnight incubation of human airway with exogenously applied MBP. This representative image shows fluorescence of immunolabeled MBP (red) in human airway following incubation with MBP 10^−5^ M overnight. Compared to lesser duration of exposure (Figure 6), longer exposure, overnight, allowed MBP greater access to cells within the media of the airway wall. Notably this longer exposure allowed for significant immunolocalization to be detected within the smooth muscle layer (green) (confocal microscopy 400x).

**Figure 8:**
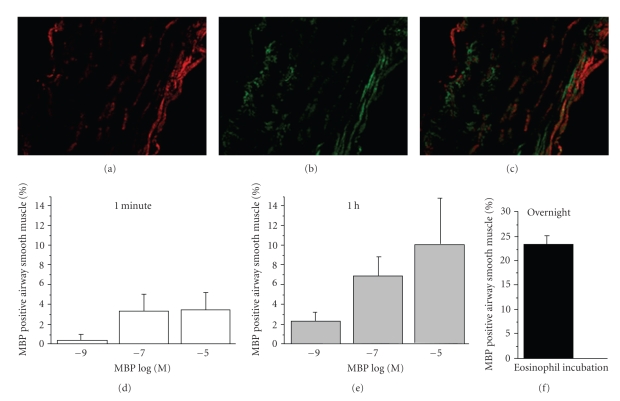
Immunolocalization of MBP in human airway following exogenous application of MBP or following incubation with eosinophils. Separate images showing smooth actin and MBP were examined. (a) shows MBP distribution (red), (b) image shows smooth muscle actin (green), and (c) image shows putting the two images together in binarized colabed images. Using the Metamorph system, the two pictures were analyzed to find areas measured in pixels. Exposure of 1 min (solid) or 1 h (light gray) as to higher concentrations of MBP allowed more MBP to access smooth muscle area compared to lower concentrations no significance was found by percentage smooth muscle with MBP in smooth muscle area. For same concentrations, longer exposure, 1 h compared to shorter exposure, 1 min, increased the percentage of smooth muscle colocalized with MBP. Additionally, substantial MBP (23.8% ± 4.9) was found in the ASM layer following overnight incubation (black) with human eosinophils (*n* = 3 determinations from 9 patients for each concentration and time).

Figure 6 shows representative images from identical experiments performed in airways from 9 distinct patients of immunolabeled MBP in human airway following incubation with MBP for 1 min and 1 h. Brief exposure, 1 min (Figure 6), resulted in limited MBP deposition to the epithelial and outer surface of the airway. However, longer exposure, 1 h, allowed some MBP to access cells within the media of the airway wall. More importantly, longer exposures allowed for immunolocalization to be detected within the smooth muscle layer. This effect was increased by greater concentrations of MBP (10^−5^ M) compared to lower concentrations of MBP (10^−7^ M). In all control tissue, smooth muscle actin was detected but no MBP content was detected (Figure 6, top panels). Compared to 1 min and 1 h duration of exposure (Figure 6), longer overnight exposure (Figure 7) allowed MBP greater access to cells within the media of the airway wall, especially within the smooth muscle layer. A summary of the time- and concentration-dependent effects of the access of MBP to the smooth muscle layer is shown in Figure 8.

### 5.8. Distribution of Immunoreactive MBP and Neuron-Specific PGP 9.5 in Human Airway Bronchus following Overnight Incubation with Human Eosinophils

**Figure 9:**
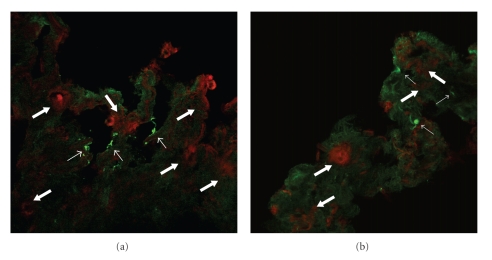
Distribution of immunoreactive MBP and nerve PGP 9.5 in human airway following overnight incubation with human eosinophils. Representative images of 2 mm human airway incubated overnight with eosinophils. Immunoreactive PGP 9.5 (green) and MBP (red) are shown. Though some eosinophils appeared near nerves (small arrows) in the human airway (a), many eosinophils, as well as extracellular MBP deposition, occurred within the airway wall not associated with airway nerves (b) (thick arrows) under higher magnification (confocal microscopy 400x).

In sections of human bronchi, eosinophils and extracellular MBP were found within airways following 18–24 h incubation with human eosinophils. Airway neurons were identified immunohistochemically by an antibody against PGP 9.5, a nonspecific neural marker for ubiquitin C-terminal hydrolase found in neuroendocrine bodies and nerves [29]. Eosinophils and extracellular MBP were identified using an antibody for MBP (data not shown). The relative area staining for PGP 9.5 with MBP was not significantly different from the relative area staining positive for MBP not containing PGP 9.5. Specifically, the relative area of PGP 9.5 positive and MBP positive was 7.0 ± 3.0 percent compared to PGP 9.5 negative and MBP positive 5.9 ± 1.3 percent, (*P* > .7, *n* = 3 determinations from 10 distinct patients). However the relative area containing eosinophils and PGP 9.5 positive neurons was significantly less than the relative non-PGP 9.5 area containing eosinophils. Specifically, the relative non-PGP 9.5 area also containing eosinophils was 2.8 ± 0.65 percent compared to only 0.70 ± 0.60 percent of the relative PGP 9.5 positive area, (*P* < .03   *n* = 3 determinations from 10 distinct patients). Thus, there was no evidence of specific recruitment of eosinophils, nor eosinophil degranulation in the area of PGP 9.5 neuronal tissue compared to the rest of the airway wall (Figure 9).

### 5.9. Eosinophils Location and Immunoreactive MBP and Eotaxin

**Figure 10:**
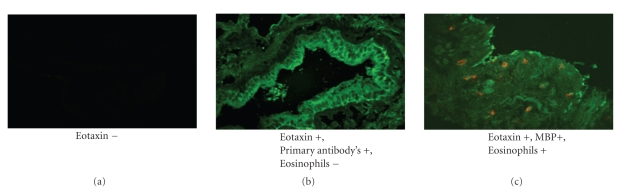
Eosinophils location and immunoreactive MBP and Eotaxin in human airway following overnight incubation with and without human eosinophils. Representative images of 2 mm human airway incubated overnight with and without eosinophils. Immunoreactive eotaxin (green) and MBP (red) are shown. (a) shows absence of primary antibodies, (b) image shows epithelium and airway wall eotaxin (green), and (c) image shows epithelium and airway wall eotaxin (green) and MBP (red) (confocal microscopy 400x).

To determine the expression of the chemokine Eotaxin in our cultures, we determined by qualitative immunofluorescent microscopy the localization of Eotaxin as well as MBP in human airways incubated 18 h with and without eosinophils. There were no apparent differences (*n* = 3 determinations from 5 distinct patients) in Eotaxin expression (Figure 10) which occurred constitutively within the bronchial epithelium and airway wall in tissues incubated both with and without eosinophils.

## 6. Discussion

Eosinophilic airway inflammation is a dominant finding in human asthma, as well as in some patients with smoking-related COPD [30]. The present study indicates that eosinophils can attach and migrate into the media of unsensitized small human airways in vitro and many of these eosinophils colocalize to airway smooth muscle cells. Furthermore, these eosinophils may degranulate in vitro allowing for analysis of MBP localization along the surfaces and within the media of the airway. Moreover, incubation of small human airways with isolated MBP reveals a distribution kinetic which is both time and concentration dependent. In addition we determined that MBP's diffusion is restricted to the airway surface during brief and low concentration incubation, but may penetrate the airway media and effect medial layer cell function, during either prolonged high-concentration incubation or during incubation with the “MBP carrier cell”, the eosinophil. This restricted diffusion distribution is likely due to MBP's large cationic charge [31].

Our findings explain the conflicting results that prior studies have produced following the application of MBP to intact airway or cultured airway cells. These prior studies have differed in that MBP's effects on airway smooth muscle may be direct [18] or indirect [10]. We [18] have previously shown that MBP directly elicits airway myocyte calcium mobilization. Others have shown that an indirect effect of MBP may be through an effect on epithelial-derived mediator release [32–34], increased epithelial permeability [35], cytotoxicity [36], or by inhibiting muscarinic M_2_ receptors [16]. These conflicting results may be due to the manner in which purified MBP is applied to tissues and cells in culture. Though the eosinophil transports MBP in a solubilized form within specialized acidified granules to prevent its precipitation and premature binding to cells [37], the application of purified MBP directly to tissues and cells is problematic. For example, experimental [10] application of high concentrations (1 mg/mL, 71 *μ*M) for brief exposure periods (15 min) likely limits access of MBP to airway surface cells, such as the airway epithelium. However, our study provides the first detailed description of the distribution and localization of MBP and human eosinophils in nonsensitized small human airways following in vitro incubation. This permitted a detailed examination of the time and concentration dependence of MBP's distribution through airway tissue.

In this study, we used surgically discarded human lung specimens to obtain third- to sixth-generation bronchi. Freshly isolated human eosinophils, as well as previously isolated eosinophil-derived MBP, were used to study the localization and distribution of both eosinophils and MBP within bronchial segments following in vitro incubation. Our data indicate that eosinophils rapidly (<10 min) adhered to human airway tissue (Figure 2(a)) as well as to both cultured human airway myocytes (Figure 2(b)) and primary cultures of airway epithelium. These results are consistent with the data by Hughes et al. [38] that freshly isolated eosinophils from healthy donors rapidly attach to ASMC in vitro, an effect that involves VCAM-1 and ICAM-1 and is modulated by TNF-alpha. In addition, though IL-5 may upregulate integrin-dependent eosinophil adhesion to airway epithelium [39], a significant eosinophil adhesion to unstimulated airway epithelium is independent of CD18/ICAM-1 [40]. We quantified eosinophil binding to unstimulated cultured airway myocytes and airway epithelium to be 10% and 20%, respectively. In our experiment all human airways incubated 18 h either with and without eosinophils expressed immunoreactive Eotaxin which was not different in airway epithelium versus within the airway media. It is likely that Eotaxin or other constitutively expressed chemokines (Rantes) and adhesion molecules (ICAM-1, VCAM-1) were likely responsible for eosinophil migration within the airway wall [41, 42]. 

Following 18–24 h incubation of bronchial segments with eosinophils, eosinophils were found to adhere to the epithelial layer as well as migrate within the airway wall and localize to areas that included the smooth muscle layer (Figures 3(a) and 3(b)). We determined that ~22% of airway wall eosinophils are juxtaposed with airway myocytes. These findings are similar to clinical studies by Haley et al. [19], Tulic et al. [20], Van [4], and Prins [5]; each of whom noted that in small human airways taken from asthmatics eosinophils were found predominately in the “outer” layer between the smooth muscle and the adventia. Though stimulated airway smooth muscle cells produce eotaxin [41, 43], a potent chemoattractant for eosinophils, little study has been done to analyze the proximity of eosinophils and airway myocytes in subjects with asthma or experimental models of asthma. Moreover, in none of these human above studies was an analysis of the extracellular deposition of granule proteins, nor a precise localization of the eosinophils in relative distance and distribution to myocytes and airway neurons. 

Following overnight incubation with eosinophils in human airway, eosinophil degranulation was evident by two patterns of extracellular MBP deposition: a broad linear deposition along both the adventitial and epithelial surfaces, and the discrete focal deposition within the airway media, including the airway myocyte layer (Figure 4). This latter pattern represented both MBP contained within eosinophils as well as discrete focal deposition found surrounding eosinophil degranulated “ghosts”. We calculated that the immunologically detected volume of distribution of MBP within the airway wall from single degranulated eosinophils is ~300 *μ*m^3^. This takes into account the diffusion of MBP away from the degranulated eosinophil, as well as the zone of clearing of MBP where the eosinophil resided (i.e., eosinophil “ghost”, Figure 5). Given the approximate content of MBP within an eosinophil as 8.98 × 10^−9^ mg (MW = 14,000) [44], the estimated concentration of MBP in these discrete deposits within the airway is approximately 2.14 × 10^−3^ M. This concentration is significantly greater than the concentration of MBP (10 *μ*M/mL, or 7.14 × 10^−7^ M) which is known to be toxic to and causes erosion of the epithelium [45]. Moreover, we have previously shown [18] that these concentrations are well within the range to alter basal, as well as agonist-elicited intracellular calcium mobilization in cultured airway myocytes. The above findings are not surprising given MBP's highly reactive sulfhydryl content allowing MBP the ability to readily aggregate with itself and other proteins [31].

Alternatively, when human airways were exposed to exogenous MBP, the distribution of MBP within the airway wall occurred strictly in a time- and concentration-dependent manner. At brief exposure times of MBP for 1 min, only limited, MBP deposition to the epithelial and adventitial surface of the airway occurred. As the exposure time lengthened to 1 hour MBP penetration to cells within the media of the airway wall occurred. Finally, following overnight incubation, MBP could be detected easily within the smooth muscle layer. This latter effect was also concentration dependent (Figures 6 and 7). Our results differ from those of a recent in vivo study [46] which indicated that in 22 patients with chronic rhinosinusitis (CRS) all tissue specimens showed intact eosinophils, and extracellular MBP deposition was found only within luminal mucus. These results suggested that eosinophils might be found beneath the sinus epithelium but only degranulate outside of tissue within mucus. However, our data support the hypothesis that eosinophil degranulation and release of MBP may occur within the airway wall and may contribute to the pathophysiology observed in asthma.

Others have suggested a role of eosinophil-derived MBP as a cause of M_2_ muscarinic receptor dysfunction. Specifically, in histologic airway sections from humans who have died from acute asthma, eosinophils are found near PGP 9.5 positive nerves [47]. Moreover, these investigators determined that, in antigen-challenged guinea pigs, MBP may allosterically bind to M_2_ muscarinics receptor thereby inducing AHR in vivo [48, 49]. In a latter study they showed that functional antagonism of MBP with the systemically administered polyanion heparin reversed this AHR [17]. Additionally, Gu et al. [50, 51] have demonstrated that MBP potentiates the excitability of neurons by inhibition of the sustained delayed-rectifier voltage-gated K+ current and the fast-inactivating K+ current. As such, MBP release near airway wall sensory neurons may elicit airway hyperresponsiveness via C-fiber afferent activation. However, current understanding suggests a minimal role of the vagal nervous system in clinical asthma. However, in neither of the above experiments did investigators take into account a direct effect of MBP at the level of the airway myocyte, a finding that we previously noted [18]. Specifically, we have shown that MBP augments airway myocyte intracellular calcium regulation. Though our experiments were conducted in vitro in non-sensitized tissue, we did not find a specific recruitment of eosinophils, nor extracellular MBP to airway neuronal loci compared to other sites within the airway wall. Thus, mechanistically sensitization may be necessarily related to eosinophil migration and recruitment specifically to sites of airway neurons.

The functional role of MBP in asthma models has recently been challenged in a sensitized ovalbumin model of asthma using mMBP-1 knockout mice [52]. Though OVA-induced increases in AHR to nonspecific stimuli were not mediated by mMBP-1, this model may not require eosinophil degranulation for observed increases in AHR, the temporal and spatial release of MBP may not elicit an AHR response, or other eosinophil-derived granule protein may be responsible for AHR. However, in other models of asthma, AHR has been shown to correlate with extracellular MBP deposition [30] including localization of eosinophils to the airway smooth muscle band [53]. In fact, neutralization of endogenous eosinophil released MBP by specific antiserum prevents bronchial hyperreactivity in antigen-challenged guinea pigs [54].

In conclusion, in nonsensitized small human airway, eosinophils adhere, migrate within the airway wall, and undergo spontaneous degranulation of MBP. We acknowledge that while the presence of COPD-related inflammatory signaling within some of our tissue specimens may have influenced the migration of eosinophils it was unlikely to influence the extracellular pattern of deposition of MBP, nor the interpretation of MBPs rather restricted diffusion and ability to deposit within the airway wall, and hence influence directly myocyte function in vivo. 

Immunohistochemically, MBP's volume of distribution is restricted to ~300 *μ*m^3^ from the degranulated eosinophil producing locally high concentrations within the smooth muscle layer nearing 10^−3^ M. Because of the restricted nature of MBP's diffusion its distribution is both time and concentration dependent, and also depends on its form of delivery, that is, within intact eosinophils or as native MBP. Brief in vitro applications of MBP, especially only to the luminal surface of the airway, may not capture the complete physiologic influence of MBP on eliciting AHR. In this in vitro model of eosinophil incubation with bronchial segments, there does not appear to be specific recruitment of eosinophils to airway neurons. Thus, our findings suggest that studies and therapeutical interventions designed specifically to the target eosinophil and MBP need to consider the spatial and concentration effects of granule proteins released within the airway wall as well as the airway lumen.

## Figures and Tables

**Table 1:** Patient characteristics and pulmonary function tests.

Patient characteristics	Number
Age (yr)*	63.8 ± 9.0
Sex (Male/Female)	10/12
Smoking pack years*	33.4 ± 13.6
	Current = 6
Smoking history	Past = 12
	Never = 4
Current corticosteroid use	2

Pulmonary function Classification of COPD	
Normal	1
Mild COPD	5
Moderate COPD	8
Severe COPD	8
Positive bronchodilator Response	3/17

*Values are means ± SD.
